# Supplementary material for: Sorting at embryonic boundaries requires high heterotypic interfacial tension
Source: Nat Commun. 2017 Jul 31;8:157. doi: 10.1038/s41467-017-00146-x (PMC5537356; doi:10.1038/s41467-017-00146-x)
Supplement: Supplementary file 2 — Supplementary Software 1 [file 41467_2017_146_MOESM2_ESM.zip › PottsModel/SrcPottsModel/doc/index-files/index-1.html]

A-Index


JavaScript is disabled on your browser.


Skip navigation links


- Overview
- Package
- Class
- Use
- Tree
- Deprecated
- Index
- Help

- Prev Letter
- Next Letter

- Frames
- No Frames

- All Classes

A B C D E F G H I J K L M N O P R S T U V W 


## A

aAutomaticMode - Variable in class engine.PottsEngine


aAutomaticMode - Variable in class gui.PottsFrame


aColor - Variable in class gui.PixelDisplay


aConstants - Variable in class model.Cell


actionPerformed(ActionEvent) - Method in class gui.PottsFrame


add(EnergyFunction.Energy, double) - Method in class engine.Utils.EnergyTracker


addCanvas(PottsCanvas) - Method in class gui.PottsFrame


addCell(Cell) - Method in class model.CellSet
:   Adds a cell to the CellSet.

addObserver(Observer) - Method in class mvc.AObservable
:   Add an observer to the observable object.

addObserver(Observer) - Method in interface mvc.IObservable
:   Add an observer to the observable object.

addOrRemovePixel(Pixel, boolean) - Method in class gui.CellDisplay


addPixel(Pixel) - Method in class model.Cell
:   Add Pixel to Cell.

addTabToSidePane(String, JPanel) - Method in class gui.PottsFrame


addToManagerStatistics(List<Statistic>) - Method in class engine.Statistic


aDeltaEnergyTracker - Variable in class engine.PottsEngine.StateVariables


adjustedBoundary() - Method in class model.Pixel
:   Checks whether this APixel is at a cell boundary i.e.

adjustHue(float) - Method in class utils.HSLColor
:   Create a RGB Color object based on this HSLColor with a different
    Hue value.

adjustLuminance(float) - Method in class utils.HSLColor
:   Create a RGB Color object based on this HSLColor with a different
    Luminance value.

adjustSaturation(float) - Method in class utils.HSLColor
:   Create a RGB Color object based on this HSLColor with a different
    Saturation value.

adjustShade(float) - Method in class utils.HSLColor
:   Create a RGB Color object based on this HSLColor with a different
    Shade.

adjustTone(float) - Method in class utils.HSLColor
:   Create a RGB Color object based on this HSLColor with a different
    Tone.

aDoMCS - Variable in class engine.PottsEngine


aDone - Variable in class engine.PottsEngine


aDoSwap - Variable in class engine.PottsEngine


aGraphicalOutputEnabled - Variable in class engine.PottsEngine


aHeight - Variable in class model.Lattice


aLattice - Variable in class engine.PottsEngine


aMCSAndPause - Variable in class gui.PottsFrame


aNumEdges - Static variable in class gui.PottsCanvas


aNumEdges - Static variable in class gui.SnapshotManager


AObservable - Class in mvc
:   Implements a class which can be extended to provide observer support.

AObservable() - Constructor for class mvc.AObservable


aPaused - Variable in class gui.PottsFrame


aPixelSwapSource - Variable in class engine.PottsEngine.StateVariables


aPixelSwapTarget - Variable in class engine.PottsEngine.StateVariables


aPottsHeight - Variable in class gui.PottsCanvas


aPottsHeight - Variable in class gui.SnapshotManager


aPottsWidth - Variable in class gui.PottsCanvas


aPottsWidth - Variable in class gui.SnapshotManager


areaElasticity - Variable in class model.Constants.ParameterManager


AreaEnergyFunction - Class in model


AreaEnergyFunction() - Constructor for class model.AreaEnergyFunction


AreaEnergyStatistic - Class in engine


AreaEnergyStatistic(PottsEngine) - Constructor for class engine.AreaEnergyStatistic


AreaStatistic - Class in engine


AreaStatistic(PottsEngine) - Constructor for class engine.AreaStatistic


AreaStatistic(PottsEngine, int) - Constructor for class engine.AreaStatistic


areaStd - Variable in class model.Constants.ParameterManager


areHorizontalNeighbours(Pixel, Pixel) - Method in class model.HexagonPositionManager


areHorizontalNeighbours(Pixel, Pixel) - Method in interface model.IPositionManager


areHorizontalNeighbours(Pixel, Pixel) - Method in class model.SquarePositionManager


areNeighbors(Pixel, Pixel) - Static method in class model.HexagonPositionManager


areNeighbours(Pixel, Pixel) - Method in class model.HexagonPositionManager


areNeighbours(Pixel, Pixel) - Method in interface model.IPositionManager


areNeighbours(Pixel, Pixel) - Method in class model.SquarePositionManager


areSwappable(Pixel, Pixel) - Static method in class model.Pixel
:   Check if resulting swap will not produce a disconnected pixel.

areVerticalNeighbours(Pixel, Pixel) - Method in class model.HexagonPositionManager


areVerticalNeighbours(Pixel, Pixel) - Method in interface model.IPositionManager


areVerticalNeighbours(Pixel, Pixel) - Method in class model.SquarePositionManager


aStateVariables - Variable in class engine.PottsEngine


aSwapAndPause - Variable in class gui.PottsFrame


aThread - Variable in class engine.PottsEngine


atLatticeEdge() - Method in class model.Pixel


aToRedraw - Variable in class engine.PottsEngine.StateVariables


attachFrame(PottsFrame) - Method in class gui.PottsCanvas


attachPlotPanel(PlotPanel) - Method in class engine.Statistic


aWidth - Variable in class model.Lattice


aX - Variable in class model.Pixel


AxialCoordinates(double, double) - Constructor for class gui.Hexagon.AxialCoordinates


aY - Variable in class model.Pixel

A B C D E F G H I J K L M N O P R S T U V W

Skip navigation links


- Overview
- Package
- Class
- Use
- Tree
- Deprecated
- Index
- Help

- Prev Letter
- Next Letter

- Frames
- No Frames

- All Classes
